# Comparative genome-wide characterization and evolutionary insights into the *AP2/ERF* gene family in three *Coffea* species (*C. canephora*, *C. eugenioides*, and *C. arabica*)

**DOI:** 10.1186/s12864-025-11850-0

**Published:** 2025-07-11

**Authors:** Sunchung Park, Ezekiel Ahn, Dapeng Zhang, Lyndel W. Meinhardt

**Affiliations:** Sustainable Perennial Crops Laboratory, United States Department of Agriculture, Agriculture Research Service, Beltsville, MD USA

**Keywords:** *Coffea arabica*, *Coffea canephora*, *Coffea eugenioides*, AP2/ERF family, Orthology, Gene duplication, Purifying selection

## Abstract

**Background:**

The APETALA2/ETHYLENE-RESPONSIVE FACTOR (AP2/ERF) transcription factor family plays a crucial role in plant development and stress responses. Characterized by the conserved AP2 DNA-binding domain, this family includes key subfamilies such as ERF, AP2, and RAV. Understanding the composition and evolutionary dynamics of the *AP2/ERF* family in coffee is essential for advancing the development of stress-resilient cultivars. Leveraging recent genomic resources for *Coffea canephora* (robusta coffee), *Coffea arabica*, and *Coffea eugenioides*, this study aims to provide a comprehensive genome-wide characterization and evolutionary analysis of the *AP2/ERF* family within these economically important species.

**Results:**

A total of 453 *AP2/ERF* genes were identified across the three *Coffea* species, constituting approximately 0.48% of their protein-coding genes. Chromosomal mapping and synteny analysis revealed a high degree of conservation among *C. canephora*, *C. arabica*, and *C. eugenioides*, reflecting their close evolutionary relationships. Gene duplication events were found to significantly contribute to the expansion of the *AP2/ERF* family, accounting for 16% of the total gene count. Subgroup IX, associated with disease resistance, exhibited substantial variation between the two parental species and their descendant subgenomes in *C. arabica*, indicating possible lineage-specific gene loss or expansion. Similarly, Subgroup III, linked to temperature tolerance, showed distinct expansion patterns among the coffee species, hinting at adaptive responses to varied environments.

**Conclusions:**

These comparative analyses of the *AP2/ERF* transcription factor family in *Coffea* species provide valuable insights into the adaptive evolution of this gene family under diverse environmental stresses. The observed subgroup-specific variations provide a basis for further functional studies and underscore potential candidates for breeding programs aimed at enhancing stress resilience and sustainability in coffee production.

**Supplementary Information:**

The online version contains supplementary material available at 10.1186/s12864-025-11850-0.

## Introduction

The *AP2/ERF * superfamily is a large group of plant-specific transcription factors known for their versatile roles in regulating developmental processes and responding to environmental stresses, particularly abiotic stresses such as drought, cold, and high salinity [1–4]. This superfamily is characterized by the presence of at least one AP2 domain, a conserved DNA-binding sequence of about 70 amino acids that allows direct binding to *cis*-elements in downstream target genes. The *AP2/ERF* superfamily can be further subdivided into subfamilies– AP2, ERF, and RELATED TO RELATED TO ABSCISIC ACID INSENSITIVE 3/VIVIPAROUS 1 (RAV)–based on the number and type of AP2 domains as well as other structural features, with each subfamily playing distinct roles in plant physiology, development, and stress responses [5–7].

Among the subfamilies, the *ERF* subfamily is the most abundant, typically consisting of members with a single AP2 domain. Genes within this subfamily are essential for activating signaling pathways involved in both biotic and abiotic stress responses, regulating processes such as hormone signaling, oxygen sensing, and metabolite biosynthesis. The *ERF* subfamily can be further divided into 11 subgroups–I, II, III, IV, V, VI, VII, VIII, IX, X, and V-L–based on a comprehensive analyses of *AP2/ERF* genes in *Arabidopsis thaliana* (hereafter *Arabidopsis*) and *Oryza sativa* (rice) [5]. Each subgroup is associated with distinct roles in stress responses. For instance, subgroup III has been extensively studied for its critical involvement in responses to low-temperature, salt, and drought stress. The *C-REPEAT BINDING FACTOR/DEHYDRATION-RESPONSIVE ELEMENT BINDING* (*CBF/DREB*) genes within this subgroup are critical for managing drought and cold tolerance, as demonstrated by functional studies in *Arabidopsis* and rice, which show their roles in activating downstream stress-responsive genes [8–10]. Another well-studied subgroup is IX, where members such as *Arabidopsis* ERF1 and tomato Pti4 are associated with defensive responses against pathogens [11, 12]. Additionally, defense-related phytohormones like ethylene, jasmonate, and salicylic acid have been shown to differentially induce the expression of group IX genes [13, 14]. In contrast, the *AP2* subfamily, featuring two tandemly arranged AP2 domains, is particularly important for organ development and morphogenesis, influencing traits like leaf architecture and flowering [15, 16]. The *RAV* subfamily, containing both an AP2 and a B3 domain, is typically involved in hormonal regulation and abiotic stress responses mediated by abscisic acid (ABA) [17, 18]. These classifications, based on sequence similarity and functional characteristics, are essential for understanding how *AP2/ERF* genes contribute to plant resilience and adaptability under various environmental conditions.

Coffee is one of the world most economically significant crops, with global trade valued at $45.5 billion in 2022 [19]. Coffee cultivation plays a vital role in the economies of many developing countries, supporting the livelihoods of over 100 million people in the value chain and underscoring its crucial contribution to global agriculture and socioeconomic development [19, 20]. Despite the rising global demand, coffee production is increasingly threatened by climate change, particularly affecting *Coffea arabica*, which accounts for 60% of global production [21, 22]. *C. arabica* belongs to the family Rubiaceae, and the order Gentianales, and is an allotetraploid species that originated from a natural hybridization event between two diploid parents, *C. canephora* and *C. eugenioides*, approximately 350–610 thousand years ago [23]. Thriving best in cooler climates with optimal temperatures ranging 18–21 °C [24–26], *C. arabica* is particularly vulnerable to rising temperatures, extreme weather, and prolonged droughts, which may severely impact yield and quality. Compounding this challenge is the relatively low genetic diversity of *C. arabica* compared to its diploid progenitors, which limits the development of new resilient cultivars essential for sustainable coffee production [27, 28].

In contrast, one of parental diploid species, *C. canephora*, which comprise 35% of global coffee production, is native to the lowland equatorial rainforests of West Africa, thrives at elevations of up to 1200 m with an annual mean temperature range of 22–26 °C [24, 25, 29]. This species exhibits greater heat-tolerance compared to *C. arabica*, making it more resilient to higher temperature environments. The other parent, *C. eugenioides*, is a wild diploid species found in highland areas at elevations of 1000–2000 m, with a mean annual temperature range of 18–23 °C [25, 30]. These differences in temperature sensitivity among *C. arabica* and its diploid progenitors highlight the genetic diversity and adaptive traits within the *Coffea* lineage.

In response to the escalating climate-related challenges, understanding the genetic basis of stress response mechanisms in coffee is critical for developing resilient cultivars and ensuring the sustainability of coffee cultivation. Transcription factors, particularly those in the *AP2/ERF* superfamily, play a pivotal role in regulating plant responses to various biotic and abiotic stresses [2, 14, 31, 32]. The recent availability of whole-genome sequences for *Coffea canephora*, *Coffea arabica*, and *Coffea eugenioides* provides an opportunity to explore the *AP2/ERF* gene family in these species, offering insights into the mechanisms underlying stress tolerance and adaptability [23, 33].

In this study, we performed a comprehensive genome-wide analysis of the *AP2/ERF* gene family across three *Coffea* species, identifying a total of 453 *AP2/ERF* genes. We assessed their chromosomal locations, phylogenetic relationships, and expression patterns. We focused on gene duplication events, calculating Ka/Ks ratios to investigate selective pressures acting on duplicated genes. Additionally, we conducted orthology analyses to explore evolutionary conservation and divergence within the *AP2/ERF* family across coffee species. Our findings underscored an important role of gene duplication in the expansion of the *AP2/ERF* gene family, revealing that lineage-specific expansion and contraction may confer adaptive advantages, especially in response to biotic and abiotic stresses. This study provides a foundation for future breeding programs aimed at developing coffee cultivars with enhanced resilience to climate stressors, supporting sustainable coffee production.

## Methods

### Identification of the *AP2/ERF* gene family in *Coffea* spp.

Protein sequences from three coffee species–*C. arabica* (GCF_003713225.1), *C. eugenioides* (GCF_003713205.1), and *C. canephora* (GCA_900059795.1)–were downloaded from the NCBI genome assembly database (https://www.ncbi.nlm.nih.gov/datasets/genome/). In cases with multiple splicing variants, the longest protein sequence for each gene was selected as a representative. For identification of the AP2/ERF family, Hidden Markov Model profiles of the AP2/ERF domain (PF00847) and B3 domain (PF02362) were obtained from the Pfam database via InterPro (https://www.ebi.ac.uk/interpro/) [34]. The AP2 domain profile was queried against the coffee protein sequences using the *hmmsearch* tool in HMMER v3.4 (http://hmmer.org). Proteins with an AP2 domain match E-value of ≤ 1e − 5 were selected, and domain presence were confirmed with *hmmscan* tool in HMMER v3.4 (http://hmmer.org). For RAV subfamily, which requires both AP2 and B3 domains, the B3 domain was further searched within the selected AP2/ERF proteins using *hmmsearch*, and proteins with an E-value of ≤ 1e-5 were classified into the RAV subfamily.

### Phylogenetic analysis of the AP2/ERF family

The identified AP2/ERF protein sequences from the three coffee species were combined and aligned using MUSCLE5 with default parameters [35]. The alignments were manually inspected and refined as needed in BioEdit [36]. Phylogenetic analysis was conducted using the neighbor-joining (NJ) method with the parameters of p-distance model, uniform rates among sites, and partial deletion for sites with less than 90% data coverage, and the Maximum Likelihood (ML) method using partial deletion for sites with less than 90% data coverage, with other parameters left at their defaults [37] in MEGA v11 [38]. The resulting phylogenetic tree was visualized in FigTree v1.4.4 (http://tree.bio.ed.ac.uk/software/figtree). For subfamily classification (AP2, ERF, RAV and Soloist), the identified AP2/ERF proteins were subjected to BLASTP searches against *Arabidopsis* AP2/ERF family proteins, which were obtained from the Plant transcription factor database (http://plntfdb.bio.uni-potsdam.de/v3.0/). Each protein was assigned to a subfamily based on the classification of its closest *Arabidopsis* homolog [5].

### Chromosomal location and gene structure analysis

The genomic coordinates of the *AP2/ERF* family genes in *C. arabica*, *C. eugenioides*, and *C. canephora* were obtained from genome annotations available in the NCBI database. Based on the coordinates in base pairs (bp), genes were mapped to their respective chromosomes. The physical locations of the genes on each chromosome were visualized using the R package *LinkageMapView* [39]. For gene structure analysis, including exon, intron, and untranslated region (UTR), structural data for each gene was retrieved from the NCBI genome database. Diagrams illustrating the exon–intron architectures were generated using a custom R script.

### Tandem and segmental duplication and ka/ks ratio analysis

To explore the contribution of gene duplications to the expansion of the *AP2/ERF* family in coffee, segmental and tandem duplications were identified. Genes within the *AP2/ERF* family were compared each other using BLASTP, and duplication types were classified based on following criteria: (1) Genes located within 1 Mb of each other with 80% or higher similarity and > 80% coverage for both query and hit genes, were considered tandem duplicates. (2) Genes meeting the similarity threshold but separated by greater than 1 Mb were identified as segmental duplicates. Chromosomal locations of segmentally duplicated genes were illustrated using the *circlize* package in the R environment [40].

To assess the selective pressure acting on duplicated genes, synonymous (Ks) and non-synonymous (Ka) substitutions rates between duplicated gene pairs were estimated. Protein sequences of each pair were globally aligned using CLUSTALW2 [41]. These protein sequence alignments were then converted to DNA alignments based on their coding sequence (CDS) using BioPerl [42]. Based on the protein and DNA alignments, Ka and Ks values along with Ka/Ks ratios were computed using *KaKs_calculator* v1.2 with a method of model-averaging [43]. The statistical significance of Ka/Ks ratios were assessed using Fisher’s exact test, with P-values of 0.05 or lower considered statistically significant.

### Orthologous relationships

To determine orthologous relationships for *ERF* subfamily genes across the coffee species and other higher plants, we included five species from the Asterid clade: *Oldenlandia corymbosa* (Rubiaceae, the same family as *Coffea*), *Catharanthus roseus* (Apocynaceae), *Daucus carota* (carrot, Apiales), *Solanum tuberosum* (potato, Solanaceae), and *Lactuca sativa* (lettuce, Asteraceae). Genomic protein datasets for these species were obtained from NCBI. The protein datasets were screened for AP2-domain containing proteins using HMMER3 with the AP2 domain profile (Pfam accession PF00847) as a query. Proteins with an AP2 domain match E-value of ≤ 1e − 5 were selected for subsequent orthologous analysis.

Orthologous clusters were generated using the OrthoFinder program v3 with default settings, which perform an all-against-all BLASTP and hierarchical clustering based on sequence similarity [44]. Before analysis, low-quality protein sequences, specifically those shorter than 20 amino acids, were removed to ensure data reliability. OrthoFinder assigned proteins into orthologous groups by comparing sequence similarity and phylogenetic relationships among the species analyzed.

### Transcript levels of *AP2/ERF* family

RNA-seq data for the three coffee species were downloaded from the Sequence Read Archive (SRA, https://www.ncbi.nlm.nih.gov/sra) using the SRA Toolkit (Supplementary Table S1). The RNA-seq reads were aligned to their respective coffee genomes using STAR v2.7.10 [45]. The resulting alignments (BAM files) were processed with *featureCounts* to obtain gene-level read counts [46]. For expression pattern analysis, the read counts were normalized to ‘counts per million’ (CPM) values and log2-transformed. Hierarchical clustering was then performed on the log2-transformed CPM values using the *hcluster* function from the R package *amap* v0.8.19.1 [47]. Clustering results were visualized using the *heatmap.2* method of the R package *gplots* v3.1.3 (https://cran.r-project.org/web/packages/gplots/index.html) [40].

## Results

### Identification of the *AP2/ERF* transcription factor family in *Coffea* species

To identify members of the *AP2/ERF* family in coffee, we utilized the protein datasets from the genomes of *C. arabica*, *C. canephora*, and *C. eugenioides.* Using hmmsearch with the AP2 domain (PF00847) as a query, we screened these genomes. This analysis identified 217 *AP2/ERF* genes in *C. arabica*, 103 in *C. canephora* and 133 in *C. eugenioides* (Supplementary Tables S2). *C. arabica* exhibited a higher number of *AP2/ERF* genes − almost double that of each diploid parent − paralleling expansions observed in other tetraploid tree species (*Salix matsudana*) [48]. The These genes represented approximately 0.49%, 0.40%, and 0.46% of the total protein-coding genes, in *C. arabica* (44,759 genes), *C. canephora* (25,574 genes), and *C. eugenioides* (29,100 genes), respectively.

**Fig. 1:**
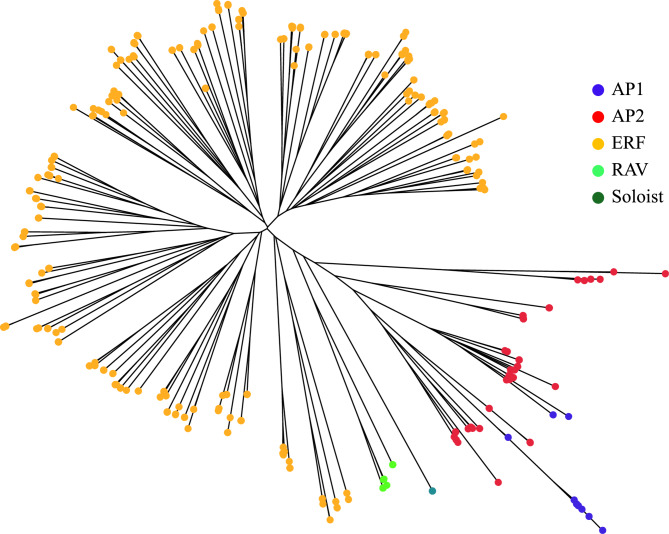
Phylogenetic analysis of the *AP2/ERF* family in *C. arabica*, *C. canephora*, *C. eugenioides.* The tree was constructed using the neighbor-joining method and includes 453 genes. Subfamilies, categorized by number of AP2 domains and presence of B3 domain, are indicated in different colors

Within the *AP2/ERF* family, members were further categorized into subfamilies based on structural features, such as the number of AP2 domains and the presence of additional DNA-binding domain B3. Proteins containing two tandem AP2 domains were classified into the AP2 subfamily, while those with a single AP2 domain were assigned to the *ERF* subfamily. Proteins featuring both AP2 and B3 domain were categorized as the *RAV* subfamily (Supplementary Table S2). To evaluate the phylogenetic relationships among the subfamilies, we constructed a phylogenetic tree using the neighbor-joining method based on protein sequence alignments. This phylogenetic analysis revealed four main clusters (Fig. 1). Among these clusters, three clusters consisted exclusively of *ERF* genes with a single AP2 domain, while the fourth cluster predominantly contained *AP2* and *RAV* genes. Notably, 15 genes across the three species–four in *C. canephora*, three in *C. eugenioides*, and eight in *C. arabica*–contained a single AP2 domain but clustered with the *AP2* subfamily genes rather than the *ERF* subfamily. These genes were thus classified as part of the *AP2* subfamily despite only one AP2 domain. In addition, four genes with a single AP2 domain did not group closely with either the *ERF* or *AP2* subfamilies. Following the classification by Nakano et al. (2006) [5], these genes were designated as *Soloist* genes. In summary, our analysis identified the *AP2/ERF* genes in each species as follows: *C. arabica* contained 36 *AP2*, 6 *RAV*, 2 *Soloist*, and 173 *ERF* genes; *C. canephora* contained 18 *AP2*, 2 *RAV*, 1 *Soloist*, and 82 *ERF* genes; and *C. eugenioides* contained 19 *AP2*, 4 *RAV*, 1 *Soloist*, and 109 *ERF* genes (Supplementary Table S2).

For ease of reference, we assigned systematic names to each *AP2/ERF* gene using a standardized nomenclature based on species abbreviations. For instance, *AP2* subfamily genes in *C. arabica* were designated with the prefix ‘CaAP2’, followed by sequential numbering (e.g., *CaAP2.1* to *CaAP2.36*). Similarly, *RAV* genes were prefixed with ‘CaRAV’ (e.g., *CaRAV.1* to *CaRAV.6*), Soloist genes with ‘CaSoloist’ (e.g., *CaSoloist.1* and *CaSoloist.2*), and *ERF* genes with ‘CaERF’ (e.g., *CaERF001* to *CaERF173*). The same naming convention was applied to *C. canephora* and *C. eugenioides*, with prefixes ‘Cc’ and ‘Ce’, respectively (Supplementary Table S2).

### Chromosomal distribution and synteny of *AP2/ERF* family genes

**Fig. 2:**
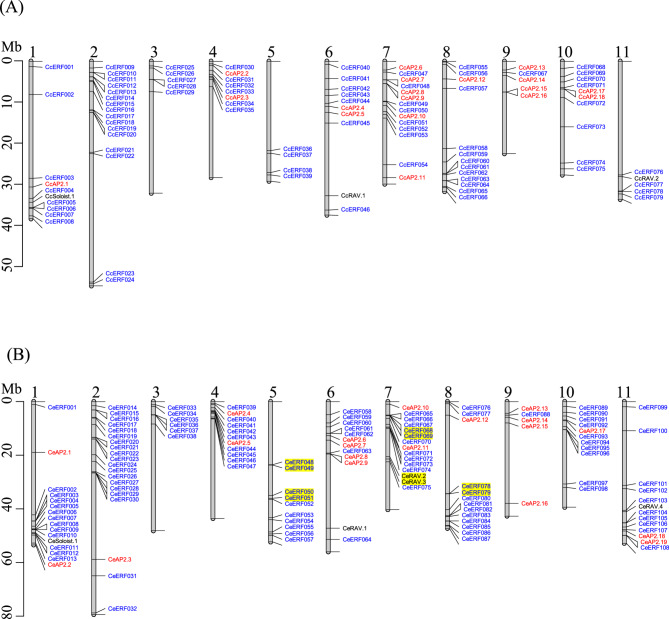
Chromosomal distribution of *AP2/ERF* genes in *C. canephora* (**A**) and *C. eugenioides* (**B**). *AP2*, *RAV*, and *Soloist* subfamily genes are represented in red, and *ERF* subfamily genes are shown in blue. Chromosome numbers are shown at the top, and chromosomal lengths are indicated on the left in megabase pairs (Mb). Tandem duplicated genes are highlighted in yellow. Genes located within 50 kb are connected, and lines at the chromosome ends mark boundaries of the chromosomes

**Fig. 3:**
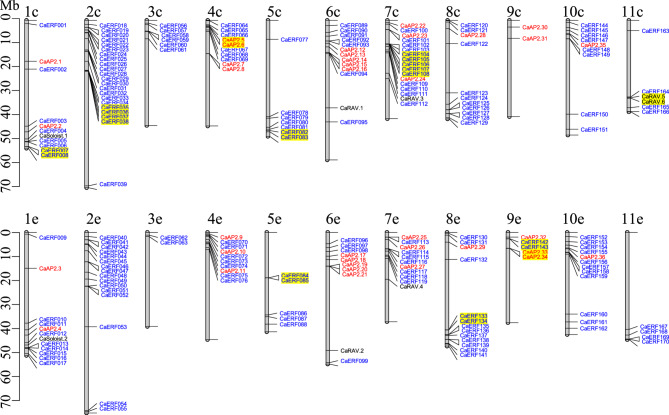
Chromosomal distribution of *C. arabica AP2/ERF *family. *AP2*, *RAV*, and *Soloist* subfamily genes are color-coded in red, and *ERF* family genes are shown in blue. Chromosome numbers are labeled at the top, with ‘c’ indicating *C. canephora* origin and ‘e’ indicating *C. eugenioides* origin. Chromosomal lengths are displayed on the left in megabase pairs (Mb). Tandem duplicated genes are highlighted in yellow. Genes located within 50 kb are connected, and lines at the chromosome ends mark boundaries of the chromosomes

To assess the chromosomal distribution of the *AP2/ERF* genes in the *Coffea* species, we mapped the genes to chromosomes of each respective species. A small subset of genes–three in *C. arabica* or *C. canephora*, and one in *C. eugenioides*–were located on non-chromosomal contigs, and these genes were excluded from this analysis (Supplementary Table S2). The mapping showed a relatively even distribution of *AP2/ERF* genes across chromosomes in *C. canephora* and *C. eugenioides* (Fig. 2), whereas *C. arabica* exhibited a more variable distribution (Fig. 3). Specifically, the number of *AP2/ERF* genes per chromosome ranging from 5 to 20 in *C. eugenioides*, 5 to 16 in *C. canephora*, and 2 to 22 in *C. arabica* (see Supplementary Table S2 for detailed locations). However, within individual chromosomes, genes tended to cluster in close proximity, resulting in uneven intrachromosomal distribution.

2

Comparative analysis of chromosomal distribution between *C. eugenioides* and *C. canephora* revealed a high degree of similarity, with gene clusters located in homologous regions on corresponding chromosomes (Fig. 2). In *C. arabica*, the distribution patterns aligned closely with those of its diploid progenitors, *C. canephora* and *C. eugenioides*, reflecting its allopolyploid origin derived from a hybridization between these two parents (Fig. 3). This high level of conservation between the diploid parents as well as between the parents and their respective subgenomes in *C. arabica* suggested that the expansion of *AP2/ERF* genes likely occurred before the divergence of these three species.

Interestingly, we observed differences in *AP2/ERF* gene numbers between the diploid parents and their descendant subgenomes. Specifically, the *C. canephora* subgenome (‘c’ chromosomes) in *C. arabica* contained 114 *AP2/ERF* genes, whereas *C. canephora* itself had 100 *AP2/ERF* genes. In contrast, the *C. eugenioides* subgenome (‘e’ chromosomes) in *C. arabica* contained 100 *AP2/ERF* genes, while *C. eugenioides* had 132 genes–a notable increase relative to its homologous gene count in *C. arabica*. This discrepancy in *AP2/ERF* gene count suggested post-hybridization gene variation, potentially driven by adaptation to environmental conditions.

**Fig. 4:**
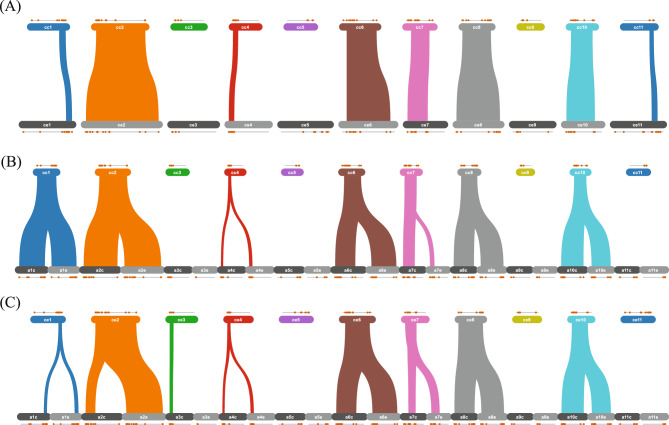
Genomic collinearity among *C. arabica*, *C. arabica, *and *C. eugenioides*. **A **Syntenic regions between *C. canephora* (cc) and *C. eugenioides* (ce) are connected by lines illustrating conserved gene order. **B** Synteny between *C. canephora* (cc) and *C. arabica* (ca.). **C** Synteny between *C. eugenioides* and *C. arabica*. Orange dots at the top of each panel represent the locations of *AP2/ERF* family genes

To further understand the evolutionary relationships and structural conservation of the *AP2/ERF* family across the three coffee species, we conducted chromosomal synteny analysis. Consistent with chromosomal distribution patterns, we observed extensive synteny between *C. canephora* and *C. eugenioides* (Fig. 4A), and between *C. arabica* and its parental species (Fig. 4B and C). Specifically, chromosome 2, 6, 7, 8 and 10 exhibited high levels of collinearity, indicating that the *AP2/ERF* genes have remained conserved in these regions since the last common ancestor of *C. canephora* and *C. eugenioides*, and even after hybridization events that gave rise to *C. arabica*.

### Duplications in the *AP2/ERF* gene family

**Fig. 5:**
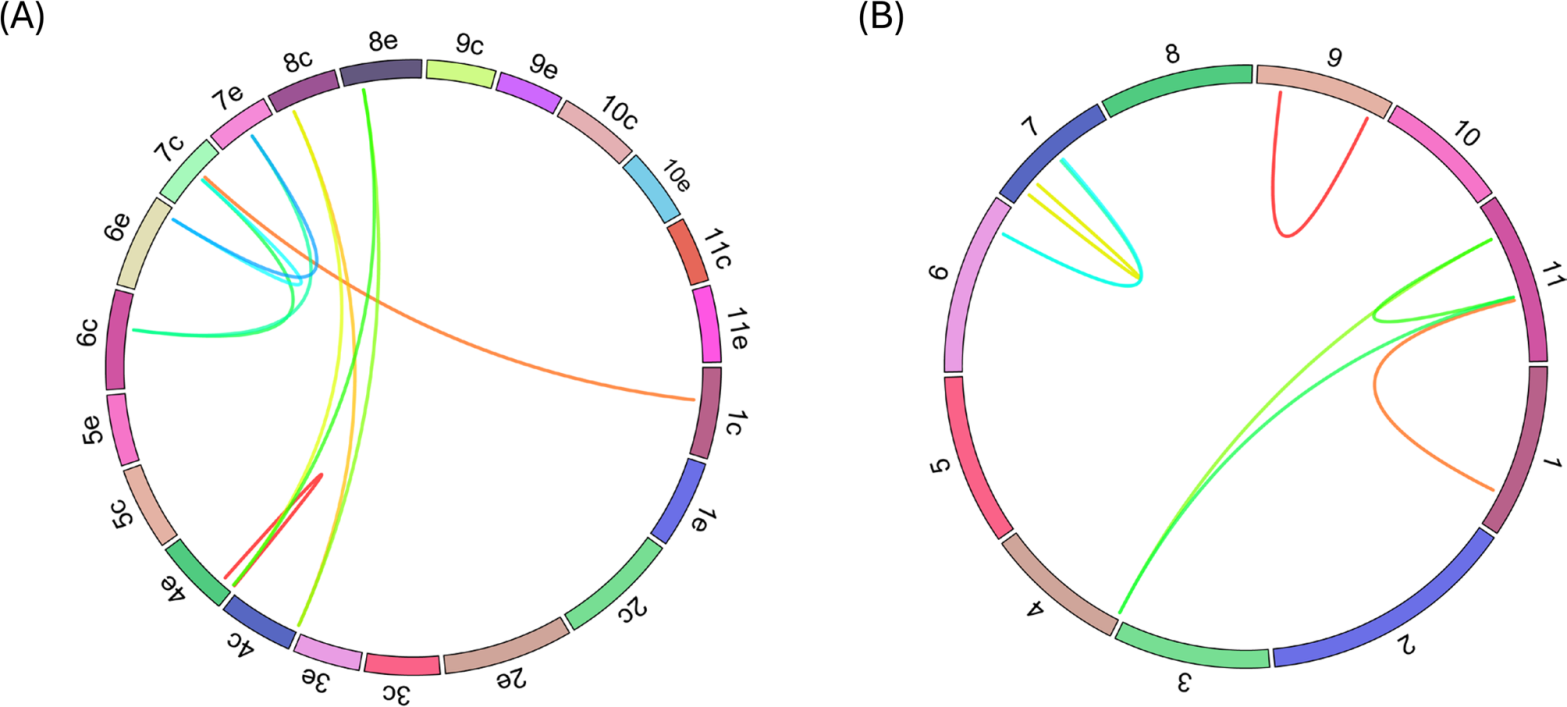
Genomic distribution of segmentally duplicated *AP2/ERF* genes in *C. arabica *(**A**) and *C. eugenioides *(**B**). Duplication events are depicted by colored lines, with each color representing a pair of duplicated genomic regions

Gene family expansion in plants often results from tandem and segmental duplications, contributing to functional diversity and adaptability [32, 49, 50]. To investigate the expansion of the *AP2/ERF* family in coffee, we analyzed gene duplication events. *C. eugenioides* showed 14 duplicated gene pairs, consisting of five tandem and nine segmental duplications. In contrast, *C. arabica* exhibited 100 duplicated gene pairs, including 88 segmental and 12 tandem duplications. Among the segmental duplications in *C. arabica*, 77 gene pairs were located on homoeologous chromosomes derived from the two parental species, suggesting that these pairs are homologous genes inherited from each parent rather than true segmental duplications. The remaining 11 pairs, found on non-homoeologous chromosomes, were classified as true segmental duplicates (Fig. 5A, Supplementary Table S3). Using the same criteria, we identified no duplicated pairs in *C. canephora*.

In *C. eugenioides*, the tandemly duplicated genes formed five clusters located on chromosomes 5, 7, and 8 (Fig. 2B; Supplementary Table S3). Chromosome 5 and 7 each contained two clusters, while chromosome 8 had one. In *C. arabica*, ten tandem duplicate clusters were identified (Fig. 3). Four of these clusters were located on *C. eugenioides-*descendant chromosomes (chromosomes, 5e, 8e, and 9e), two of which also found in *C. eugenioides* (chromosome 5 and 8), suggesting that these duplicates were likely inherited from *C. eugenioides* (Figs. 2B and 3). However, the presence of two unique clusters on *C. arabica* (chromosome 9e), absent in *C. eugenioides*, suggested post-hybridization duplication in *C. arabica* or possible gene loss in *C. eugenioides*. Additionally, three tandem clusters present in *C. eugenioides* (on chromosomes 5 and 7) were not found on homologous chromosomes 5e and 7e in *C. arabica* (Figs. 2B and 3), indicating possible *C. eugenioides* lineage-specific duplication or post-hybridization gene loss in *C. arabica*. Furthermore, six tandem duplications found on *C. canephora-*descendant chromosomes in *C. arabica* (1c, 2c, 4c, 5c, 7c, and 11c) were not observed in *C. canephora*, suggesting that these duplications might be unique in *C. arabica* or lost in the *C. canephora* lineage (Figs. 2A and 3).

For segmental duplications, *C. eugenioides* displayed three clusters duplicated within the same chromosome (7, 9, and 11) and four clusters duplicated across different chromosomes (Fig. 5A). In contrast, *C. arabica* had one segmentally duplicated cluster within a single chromosome (4e) and nine clusters duplicated across chromosomes (Fig. 5B). These findings underscored the complex evolutionary history of *AP2/ERF* genes in coffee, with both tandem and segmental duplications contributing to gene family expansion, and possible lineage-specific duplication or gene loss.

Exon-intron structures provide valuable insights into gene evolution, as intron presence and number potentially influence gene regulation and functional specialization. We examined the exon-intron structures of the *AP2/ERF* family genes to assess their structural diversity and evolutionary implications. We observed distinct patterns among the subfamilies. All genes in the *AP2* and *Soloist* subfamilies contained introns, with intron numbers ranging from 3 to 10 in *C. arabica* (Fig. S1), 5 to 9 in *C. eugenioides* (Fig. S2), and 2 to 10 in *C. canephora* (Fig. S3). Conversely, the *ERF* subfamily mostly lacked introns, consistent with previous findings [5, 32]. However, some *ERF* genes contained introns, suggesting functional diversification within the subfamily. Specifically, 35% of *ERF* subfamily in *C. canephora* (28 genes) contained one to four introns, while 26% in *C. arabica* (44 genes) and 22% in *C. eugenioides* (24 genes) also contained introns, with counts similarly ranging from one to four per gene.

### Divergence rate of the *AP2/ERF* genes

Gene duplication often relaxes selective pressure on the duplicated copies, allowing for subfunctionalization through mutation, or alternatively maintaining gene function due to dosage effects [49, 50]. To explore the evolutionary selection acting on the duplicated *AP2/ERF* genes in coffee, we analyzed nonsynonymous (Ka) and synonymous (Ks) substitution ratios for duplicated gene pairs. A Ka/Ks ratio greater than 1 suggests positive selection, potential for novel functions; a ratio near 1 indicates neutral selection, implying random changes; and a ratio less than 1 is indicative of purifying selection, where selection pressure conserves original function by minimizing mutations. In *C. arabica*, Ka/Ks ratios for duplicated *AP2/ERF* gene pairs ranged from 0.166 to 0.517, with an average of 0.365. In *C. eugenioides*, Ka/Ks values ranged from 0.109 to 0.492, with an average of 0.314 (Supplementary Table S4). Both species consistently showed Ka/Ks ratios below 1, strongly suggesting that purifying selection is the predominant force acting on these duplicated genes, thereby limiting post-duplication divergence.

### Phylogenetic analysis of the *ERF* subfamily genes

**Fig. 6:**
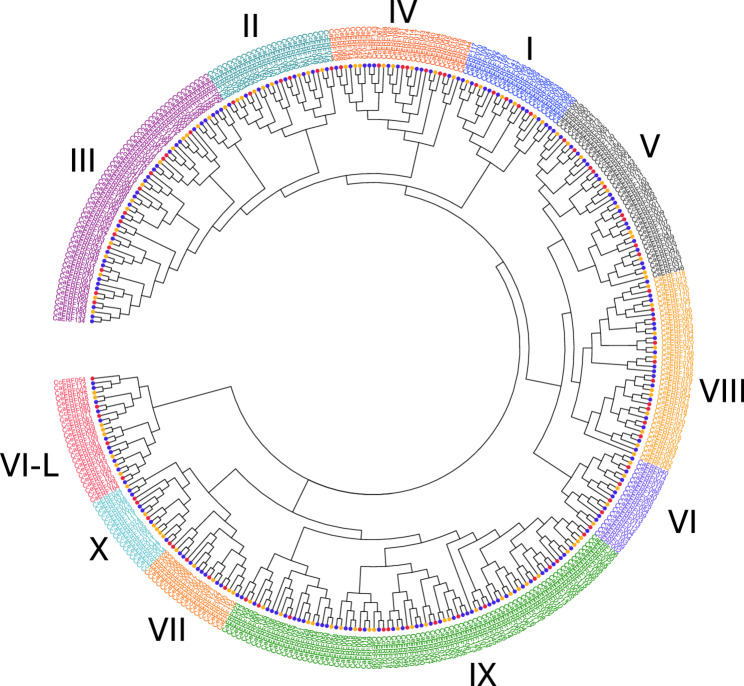
Phylogenetic analysis of 362 *ERF* genes from *C. canephora *(81 genes), *C. eugenioides* (109 genes), and *C. arabica * (172 genes). The tree was constructed using the NJ method and illustrates different subgroups, each represented by different colors. Species are depicted by colored circles at the tips of the branches: red for *C. canephora*; blue for *C. arabica*; and brown for *C. eugenioides*

The *ERF* subfamily can be further classified into functional groups, as illustrated in *Arabidopsis* [5] and other higher plants [32, 48, 51–53], where specific subgroups are associated with distinct biological roles. To determine subgroups, we employed a two-step approach combining sequence similarity and phylogenetic analyses. Initially, we used BLASTP to identify the closest *Arabidopsis* genes for each *Coffea* gene, assigning each gene to the subgroup of its closest *Arabidopsis* match (Supplementary Table S5). We then constructed a neighbor-joining (Fig. 6) and maximum likelihood (Fig. S4) phylogenetic tree to confirm and refine subgroup assignments. Both trees showed consistent subgroup classification although discrepancies were observed in the relationships among the subgroups. Most genes assigned to the same subgroup by BLASTP, formed distinct clusters in the trees. However, a few genes displayed phylogenetic placements differing from their initial subgroups. For instance, five *Coffea* genes that initially aligned with *Arabidopsis* group V or VII genes but clustered with groups VI–L genes in the NJ tree. Based on their tree positions, these genes were reclassified as group VI-L (Supplementary Table S5).

The distribution of *ERF* genes across subgroups was largely conserved among the three coffee species. Group IX was the largest subgroup, with 81 genes across the three species (42 in *C. arabica*, 14 in *C. canephora*, and 25 in *C. eugenioides*), followed by group III with 56 genes (28 in *C. arabica*, 16 in *C. canephora*, and 12 in *C. eugenioides*). The smallest subgroup, X, contained only 16 genes (6 in *C. arabica*, 6 in *C. canephora*, and 4 in *C. eugenioides*). These subgroup sizes are consistent with observation in *Arabidopsis* and *Lactuca sativa* (lettuce), where group IX is also the largest, followed by group III (Supplementary Table S6).

Interestingly, subgroup IX displayed the most notable differences in gene counts when comparing parental species with descendant subgenomes in *C. arabica*. In *C. arabica*, the subgroup IX consisted of 26 genes from the *C. canephora* subgenome (chromosomes ‘c’), compared to 14 IX genes in *C. canephora* itself (Supplementary Table S6). Similarly, while *C. eugenioides* subgenomes (chromosomes ‘e’) contained 14 IX genes, *C. eugenioides* had 25. This discrepancy suggested lineage-specific expansion or contraction within subgroup IX, which is associated with disease resistance functions [5, 54, 55]. Such variation may reflect adaptive responses to diseases unique to each species.

Subgroup III also showed considerable differences between the two diploid parents (*C. canephora*: 12 genes, *C. eugenioides*:16 genes) and between the descendant subgenomes in *C. arabica* (subgenome C = 11 genes, E = 17 genes) (Supplementary Table S6). Since group III is primarily associated with temperature tolerance [5, 10, 56, 57], these variations may contribute to differences in optimal growth temperatures among the species. The larger number of subgroup III genes in *C. eugenioides* and its corresponding ‘e’ subgenome in *C. arabica* suggested that this gene set might support adaptation to cooler temperatures.

### Orthology analysis of *ERF* subfamily genes across higher plant species

**Fig. 7:**
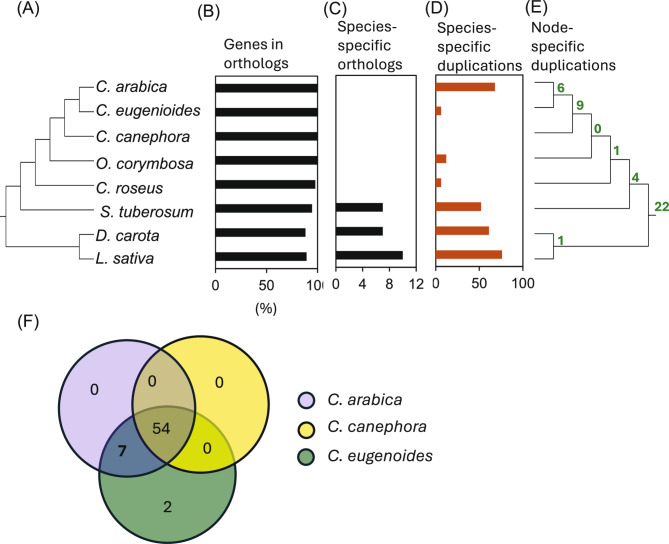
OrthoFinder analysis of *AP2/ERF* family in *Coffea arabica*, *Coffea canephora*, and *Coffea eugenioides*. **A** Species tree constructed based on all orthologous genes. **B** Percentage of genes assigned to orthogroups. **C** Number of species-specific orthogroups. **D** Gene duplication events in each species. **E** Duplication events on the internal nodes of the species tree. **F** Venn diagram comparing orthologs among the three coffee species

To investigate orthologous and paralogous relationships of the *ERF* genes in coffee and to identify potential lineage-specific expansions or contractions, we selected five representative species from the Asterid clade for comparison (Supplementary Table S7). This selection included two species within the order Gentianales–*Oldenlandia corymbosa* (Rubiaceae) and *Catharanthus roseus* (Apocynaceae)–which share a closer evolutionary relationship with *Coffea*. Additionally, we included *Solanum tuberosum* (potato; Solanales, Solanaceae), *Daucus carota* (carrot; Apiales, Apiaceae), and *Lactuca sativa* (lettuce; Asterales, Asteraceae). To establish orthologous relationships among these eight species, including the three coffee species, we employed OrthoFinder with their respective *ERF* protein datasets (*n* = 1075). ‘Species Tree from All Genes’ (STAG) method of OrthoFinder produced a species tree (Fig. 7A) consistent with established phylogenetic relationships [58–60], confirming *O. corymbosa* as the closest relative to *Coffea*, followed by *C. roseus* and *S. tuberosum*, while *D. carota* and *L. sativa* formed a separate clade. This phylogenetic arrangement supported our orthology analysis, providing a reliable framework for exploring gene conservation and divergence.

OrthoFinder identified 87 orthologous groups (Supplementary Table S8), assigning 95% (1022) of genes to an orthogroup, including all *ERF* genes in the three coffee species and *O. corymbosa*. High orthogroup assignments were also observed in *C. roseus* (98%) and *S. tuberosum* (95%), with slightly lower proportions in *D. carota* and *L. sativa* (88%) (Fig. 7B). Interestingly, species-specific orthologs were detected only in *S. tuberosum*, *D. carota*, and *L. sativa* (Fig. 7C). Among the coffee species, substantial gene duplication was observed only in *C. arabica* (*n* = 68), consistent with its tetraploid origin (Fig. 7D). In terms of ancient duplications, we identified nine duplications in the last common ancestor of the three coffee species, and six duplications in the lineage leading to *C. eugenioides* and *C. arabica* (Fig. 7E). While no orthologs were unique to each coffee species, we identified two orthologs (OG0000032, OG0000068) specific to the *Coffea* genus, indicating a low level of lineage-specific gene expansion within this genus (Fig. 7F, Supplementary Table S8).

Overall, of the 362 *ERF* genes assigned to orthologous groups, 85% (308 genes) of *Coffea ERF* genes had orthologs in both *O. corymbosa* and *C. roseus*, reflecting their shared evolutionary heritage within the Gentianales. Additionally, 79% (286 genes) of *Coffea ERF* genes had orthologs across all analyzed species, highlighting a high level of conservation of *ERF* genes likely inherited from a common ancestor. This widespread conservation suggested that the *AP2/ERF* family plays fundamental roles in plant stress responses that have been retained across diverse evolutionary lineages.

### Expression profiling of *AP2/ERF* genes

We examined the expression of *AP2/ERF* genes in coffee using public RNA-seq data. FeatureCounts analysis revealed that in *C. arabica*, 100 genes (46%) were expressed in leaf and bean tissues (Supplementary Table S9), while 58 genes (56%) in *C. canephora* (Supplementary Table S10) and 64 genes (47%) in *C. eugenioides* (Supplementary Table S11) were detected in leaf tissue. In *C. arabica*, 48% (55 out of 114) of expressed genes were from the *C. canephora* subgenome, and 44% (44 out of 100) from the *C. eugenioides* subgenome, indicating a slight expression dominance of the *C. canephora* subgenome.

Given the observed duplication events among coffee *AP2/ERF* genes, we investigated whether subfunctionalization occurred through differentiated expression patterns (Fig. S5). Most duplicated gene pairs showed similar expression profiles; however, a few pairs exhibited notable differences. For instance, *CaERF070* and *CaERF072*, as well as *CaERF082* and *CaERF083* showed moderately differentiated expression patterns, suggesting possible functional specialization following duplication.

## Discussion

Climate change pose significant challenges to sustainable coffee production, with *C. arabica* being particularly vulnerable to rising temperatures. *C. arabica* thrives within a narrow optimal temperature range of approximately 18–21 °C [24, 25], making it highly sensitive to temperature increases. Moreover, warmer climates create more favorable conditions for pathogens and pests, thereby escalating disease risks for coffee plants. For instance, coffee leaf rust (*Hemileia vastatrix*), a major fungal disease affecting *C. arabica*, proliferates more rapidly under elevated temperatures and fluctuating weather patterns [61, 62]–conditions that are becoming increasingly common due to climate change. The *AP2/ERF* superfamily of transcription factors plays a crucial role in plant responses to various abiotic and biotic stresses. Although extensive studies in other crops have demonstrated that genetic variation within *AP2/ERF* genes contributes to phenotypic diversity and stress tolerance, a comprehensive understanding of this gene family in *Coffea* species remains limited.

With the availability of genome sequencing data for coffee, we conducted a comprehensive genome-wide analysis of *AP2/ERF* genes across three *Coffea* species (*C. arabica*, *C. canephora*, *C. eugenioides*). This study identified a total of 453 *AP2/ERF* genes, representing approximately 0.46% of the total coding genes in the *Coffea* genomes (Supplementary Table S3). A previous study involving 10 plant species reported that the proportion of *AP2/ERF* genes varies, ranging from 0.43% in *Medicago* to 0.77% in artichoke, likely due to differences in gene duplication events [32]. For instance, in lettuce, *AP2/ERF* genes account for 0.59% of total protein-coding genes, with duplication events comprising 21% (48 genes) of its *AP2/ERF* family [32]. In comparison, the coffee species have relatively fewer *AP2/ERF* genes, with fewer duplication events observed in *C. arabica* and *C. eugenioides*. Specifically, duplication events account for 18% (19 genes) and 17.5% (38 genes) of the *AP2/ERF* family in *C. arabica* and *C. eugenioides*, respectively. These findings suggest that while gene duplication has played a substantial role in the expansion of the *AP2/ERF* gene family in *Coffea*, the lower frequency of duplications may contribute to the smaller proportion of this family in coffee genomes. Consistently, *C. canephora* did not exhibit any duplication events, which may account for its comparatively lower number of *AP2/ERF* family genes among the three coffee species.

Interestingly, in *C. arabica*, we identified 13 duplication events within the *C. canephora-*descendant subgenome (chromosomes labeled ‘c’), none of which were present in *C. canephora* itself. This observation suggests that these duplications are specific to the *C. arabica*-lineage, occurring after hybridization, or alternatively, they may have been lost in the *C. canephora* lineage. For example, when examining orthologous genes within subgroup IX (Supplementary Table S6), we found substantial discrepancies between the two progenitor species and their descendent subgenomes. *C. canephora* had 14 subgroup IX genes, while the *C. canephora*-descendent subgenome in *C. arabica* contained 24 subgroup IX genes. We identified four duplication events within subgroup IX of *C. arabica*, accounting for eight subgroup IX genes (*CaERF035-038*, *CaERF104-106*, *CaERF108*). This indicated that *C. canephora*- descendent genes underwent expansion through duplications, specifically in subgroup IX within *C. arabica*.

However, phylogenetic analysis suggests an alternative explanation: potential gene loss in the *C. canephora* lineage. Ideally, in the absence of duplication or loss, orthologous groups from the three coffee species should consist of one gene from each progenitor and two genes from *C. arabica* (as it is a hybrid of the two progenitors). However, the NJ tree constructed using subgroup IX genes illustrated several subclades containing only genes from *C. arabica* and *C. eugenioides* (highlighted in blue at Fig. S6). The absence of *C. canephora* counterparts in these subclades implies that orthologous genes were lost in the *C. canephora* lineage. Moreover, the duplications observed in the *C. canephora*-descendent subgenome suggest that these orthologous subgroup IX genes expanded further in *C. arabica*, unlike in *C. canephora*. Interestingly, subgroup IX genes are associated with disease-resistance [5, 54]. The expansion of these genes in *C. arabica* and their contraction in *C. canephora* may reflect evolutionary adaptations to different environmental stresses, such as disease. These findings highlight the importance of lineage-specific gene duplication and loss in shaping the *AP2/ERF* gene family in coffee species.

Gene duplication is a well-established mechanism driving genetic variation and evolution, often leading to subfunctionalization or neofunctionalization of duplicated genes. Although duplicated genes may initially have redundant functions, the accumulation of mutations over time can promote divergence. However, when duplicated genes play critical roles in essential biological processes–such as responses to abiotic stress–they are often preserved through purifying selection [63–65]. Our Ka/Ks ratio analysis supports the presence of purifying selection acting on the duplicated *AP2/ERF* genes, with ratios ranging from 0.10 to 0.52, significantly lower than 1 (Supplementary Table S3). This indicates strong selective pressure constraining the divergence of these genes and preserving their ancestral functions. The maintenance of multiple copies of essential genes may offer advantages in stress tolerance. For instance, the dosage effect observed in *Arabidopsis CBF* genes showed that freezing tolerance was impaired proportionally to the number of mutated *CBF* genes [9, 66, 67]. Similarly, the conserved duplicated *AP2/ERF* genes in coffee species may enhance their ability to cope with environmental stresses. By maintaining these duplicated genes under purifying selection, *Coffea* species could retain robust response mechanisms against abiotic stresses.

While selective constraints on protein sequences limit gene divergence, duplicated genes can acquire novel functions through altered gene expression. Promoter modifications, for instance, can lead to spatial or temporal expression divergence. This phenomenon was observed in lettuce, where duplicated genes exhibited differentiated expression. Specifically, *LsERF028/LsCBF1* and *LsERF057/LsCBF4* displayed strong activation in response to salt stress, whereas their duplicated paralogs were primarily responsive to cold stress [10]. These patterns suggested that promoter differentiation contributed to functional divergence through altered expression pattern.

In *C. arabica*, we found that duplicated genes *CaERF072-CaERF070* and *CaERF082-CaERF083* exhibited moderate expression differences across leaf, immature bean, and mature bean tissues (Fig. S4), suggesting subfunctionalization through promoter divergence. The biological importance of this evolutionary mechanism has been extensively studied in *Arabidopsis*. In *Arabidopsis*, *DREB2* (subgroup VI) and *CBF/DREB1* (subgroup III) genes exhibit a high level of protein sequence similarity and regulate the same target genes by binding to identical the *DRE/CRT cis*-element. However, *DREB2* genes primarily respond to drought stress, while *CBF/DREB1* genes respond to cold stress, allowing for fine-tuned regulation in response to different environmental stimuli [9, 56].

*C. arabica* and *C. eugenioides* thrive in cooler climates with optimal temperature ranges of 18–23 °C, whereas *C. canephora* prefers warmer climates (24–30 °C) [24, 29]. Developing temperature-resilient cultivars is essential for sustainable coffee production, especially for *C. arabica*, which is vulnerable to heat. Subgroup III of the *AP2/ERF* gene family, associated with temperature stress responses, is of particular interest. Notably, the well-known cold-responsive transcription factors (CBFs) belong to this subgroup [10, 68]. Our analysis revealed substantial differences in the number of subgroup III *AP2/ERF* genes among the three species: *C. arabica* and *C. eugenioides* possess 16 and 28 subgroup III genes, respectively, while *C. canephora* has only 12 genes. These differences likely contribute to the varying temperature tolerances among the species. Phylogenetic analysis identified genes unique to *C. arabica* and *C. eugenioides*, including subclades *CeERF094–096*, *CaERF095*, *CaERF148–149*, and *CaERF156–158* (highlighted in blue at Fig. S7), which may be linked to cooler climate adaptation. Conversely, the absence of these genes in *C. canephora* might contribute to its greater tolerance to higher temperatures. This observation of potential gene loss in *C. canephora* suggests that *C. canephora* may have initially possessed a similar range of temperature adaptability but subsequently lost certain genes through adaptive evolution to higher temperatures. While no *C. canephora*-specific genes were found in subgroup III, two subclades lacked *C. canephora-*descendant genes from *C. arabica* (highlighted in yellow at Fig. S7), suggesting that the loss of these genes in *C. arabica* may have a role in its sensitivity to high-temperatures. However, the functional impact of lineage-specific gene loss and retention on stress tolerance remains to be fully elucidated and will require approaches such targeted-gene editing, introgression, and genetic engineering. Association mapping in diverse populations may further clarify how these loci influence species-specific stress responses. Nonetheless, these lineage-specific *AP2/ERF* genes could be potential targets for improving temperature resilience and expanding coffee cultivation regions, potentially through transferring adaptive genes between species.

## Conclusions

In conclusion, this study offers valuable insights into the evolutionary dynamics of the *AP2/ERF* gene family in *Coffea* species. Our analysis revealed gene loss and retention events following the hybridization of *C. canephora* and *C. eugenioides*, leading to species-specific subclades linked to traits such as temperature tolerance and disease resistance. The observed lineage-specific expansions and contractions suggest the adaptive evolution of this gene family in response to biotic and abiotic stresses. These findings enhance our understanding of adaptive stress tolerance in *Coffea* and provide potential candidate genes that could be explored further through molecular breeding and genetic engineering to develop climate-resilient coffee cultivars.

## Supplementary Information

Supplementary Material 1.

Supplementary Material 2.

## Data Availability

All data generated during this study are included in this published article and its supplementary information files. The genomic reference datasets used for analysis were obtained from NCBI and include the following accessions: Coffea arabica (GCF_003713225.1), Coffea eugenioides (GCF_003713205.1), Coffea canephora (GCA_900059795.1), Oldenlandia corymbose (GCA_949775105.1), Catharanthus roseus (GCA_024505715.1), Solanum tuberosum (GCF_000226075.1), Daucus carota (GCF_001625215.1), and Lactuca sativa (GCF_002870075.3). RNA-seq data used in this study were sourced from the following NCBI projects: PRJNA353111 (C. canephora), PRJNA448416 (C. arabica), and PRJEB7565 (C. eugenioides).
