# Supplementary material for: Comparative genome-wide characterization and evolutionary insights into the AP2/ERF gene family in three Coffea species (C. canephora, C. eugenioides, and C. arabica)
Source: BMC Genomics. 2025 Jul 11;26:653. doi: 10.1186/s12864-025-11850-0 (PMC12247349; doi:10.1186/s12864-025-11850-0)
Supplement: Supplementary file 2 — Supplementary Material 2. [file 12864_2025_11850_MOESM2_ESM.docx]

**Comparative genome-wide characterization and evolutionary insights into the *AP2/ERF* gene family in three *Coffea* species (*C. canephora*, *C. eugenioides*, and *C. arabica*)**

Sunchung Park^1*^, Ezekiel Ahn^1^, Dapeng Zhang^1^, Lyndel W. Meinhardt^1^

^1^Sustainable Perennial Crops Laboratory, United States Department of Agriculture, Agriculture Research Service, Beltsville, MD, United States.

**Supplementary Figures S1-7**

**Figure S1.** Gene structure of *C. arabica* AP2/ERF proteins.

**Figure S2.** Gene structure of *C. eugenioides* AP2/ERF proteins.

**Figure S3.** Gene structure of *C. canephora* AP2/ERF proteins.

**Figure S4.** Phylogenetic analysis of 362 ERF genes from *C. canephora* (81 genes), *C. eugenioides* (109 genes), and *C. arabica* (172 genes).

**Figure S5.** Expression profiles of duplicated *AP2/ERF* genes in *C. arabica*.

**Figure S6.** Phylogenetic analysis of subgroup IX *AP2/ERF* genes in *C. arabica*, *C. canephora*, and *C. eugenioides*.

**Figure S7.** Phylogenetic analysis of subgroup III *AP2/ERF* genes in *C. arabica*, *C. canephora*, and *C. eugenioides*.

**Fig. S1.** Gene structure of *C. arabica* AP2/ERF proteins. In the illustrated gene models, exons are depicted by blue boxes; untranslated regions (UTR) are shown in light blue; and introns are shown as black lines.

**Fig. S2. Gene structure of *C. eugenioides* AP2/ERF proteins.** In the illustrated gene structures, exons are depicted by blue boxes; untranslated regions (UTR) are shown in light blue; and introns are shown as black lines.

**Fig. S3. Gene structure of *C. canephora* AP2/ERF proteins.** In the illustrated gene structures, exons are depicted by blue boxes; untranslated regions (UTR) are shown in light blue; and introns are shown as black lines.

III

II

I

IV

V

VIII

VI

IX

VII

X

VI-L

**Fig. S4. Phylogenetic analysis of 362 *ERF* genes from *C. canephora (*81 genes), *C. eugenioides* (109 genes), and *C. arabica* (172 genes).** The tree was constructed using the Maximum Likelihood (ML) method and illustrates different subgroups, each represented by different colors. Species are depicted by colored circles at the tips of the branches: red for *C. canephora*; blue for *C. arabica*; and brown for *C. eugenioides*.

**Fig. S5. Expression profiles of duplicated *AP2/ERF* genes in *C. arabica*.** Each row represents a gene, and each column represents a specific tissue condition. Expression levels are indicated by a red-green color gradient, with red representing high expression and green representing low expression (log2 [Counts Per Million]).

*
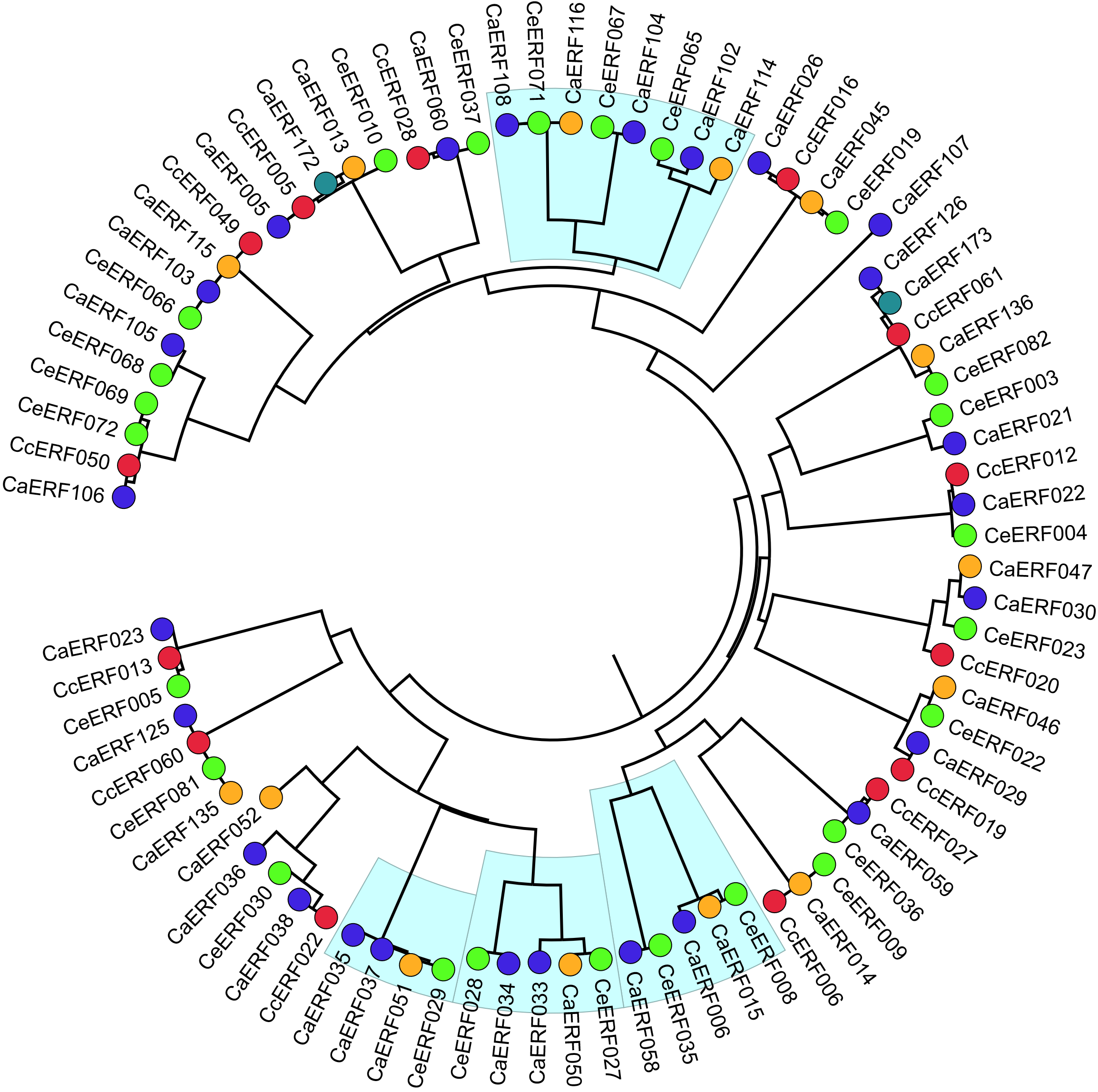
*

*C. canephora*

*C. eugenioides*

‘C’ subgenome

‘E’ subgenome

Non-chromosomal contig

**Fig. S6. Phylogenetic analysis of subgroup IX *AP2/ERF* genes in *C. arabica*, *C. canephora*, and *C. eugenioides*.** The tree was constructed using the NJ method. Circles at the tips of the branches indicates the species origin and subgenomes inherited from *C. canephora* (‘C’ chromosomes) and *C. eugenioides* (‘E’ chromosomes). Subclades lacking *C. canephora* orthologs are highlighted in blue. This figure illustrates the evolutionary relationships and gene retention patterns of subgroup IX *AP2/ERF* genes across the three coffee species, highlighting lineage-specific gene losses and expansion.

*C. canephora*

*C. eugenioides*

‘C’ subgenome

‘E’ subgenome


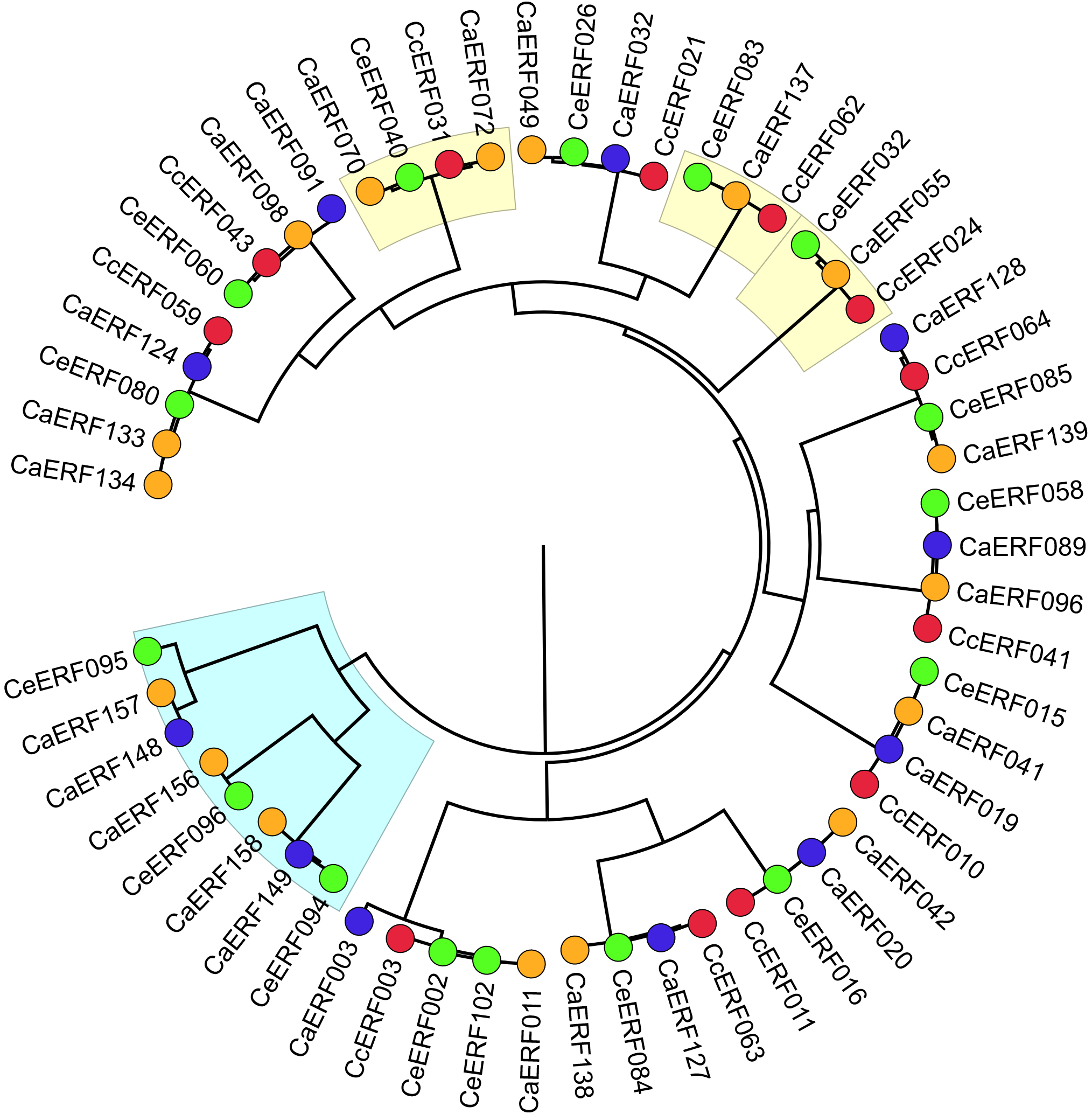


**Fig. S7. Phylogenetic analysis of subgroup III *AP2/ERF* genes in *C. arabica*, *C. canephora*, and *C. eugenioides*.** The tree was constructed using the NJ method. Circles at the tips of the branches indicates the species origin and subgenomes inherited from *C. canephora* (‘C’ chromosomes) and *C. eugenioides* (‘E’ chromosomes). Subclades lacking *C. canephora* orthologs are highlighted in blue, while those lacking ‘C’ subgenome origin are highlighted in yellow.
